# Childhood trauma and anger in adults with and without depressive and anxiety disorders

**DOI:** 10.1192/j.eurpsy.2022.664

**Published:** 2022-09-01

**Authors:** N. De Bles, L. Putz, N. Rius-Ottenheim, A. Van Hemert, E. Giltay

**Affiliations:** Albinusdreef 2, Department Of Psychiatry, Leiden, Netherlands

**Keywords:** Childhood Trauma, Anger, Depression, Anxiety

## Abstract

**Introduction:**

Childhood trauma is associated with an increased risk of anxiety and depressive disorders, but its association with anger, irritability, and related constructs has received less attention.

**Objectives:**

We aimed to investigate (1) the relationship between childhood trauma and anger constructs in adulthood, and (2) which types of childhood trauma is most predictive.

**Methods:**

In the Netherlands Study of Depression and Anxiety (NESDA), childhood trauma at baseline was assessed with a semi-structured interview. Childhood trauma was analyzed in relation to the Spielberger Trait Anger Subscale (STAS), the Anger Attacks Questionnaire, and the cluster B personality traits part of the Personality Disorder Questionnaire 4 (PDQ-4), measured at 4-year follow-up, using analysis of covariance (ANCOVA) and multivariable logistic regression analyses, adjusting for sex, age, level of education, BMI, smoking, alcohol dependency/abuse, disorder status.

**Results:**

Participants were on average 42.1 years (SD = 13.1), and 66.3% (n = 1.508) were female. Childhood trauma showed a dose-response association with all anger constructs. Zooming in, emotional neglect, and psychological, and physical abuse were associated with all anger constructs, independently of depression or anxiety. Additionally, sexual abuse and childhood life events were associated with trait anger and borderline personality traits, and trait anger and antisocial personality traits retrospectively.

**Conclusions:**

Childhood trauma is linked with anger in adulthood. Childhood trauma may cause not only anxiety and depression, but also anger, and tailored interventions (at both childhood trauma and anger itself ) might help to improve unsatisfactory relationships and prevent violent behaviors.

**Disclosure:**

No significant relationships.

